# A novel *HSF4 *gene mutation (p.R405X) causing autosomal recessive congenital cataracts in a large consanguineous family from Pakistan

**DOI:** 10.1186/1471-2350-9-99

**Published:** 2008-11-11

**Authors:** Naheed Sajjad, Ingrid Goebel, Naseebullah Kakar, Abdul Majeed Cheema, Christian Kubisch, Jamil Ahmad

**Affiliations:** 1 Faculty of Biotechnology and Informatics, BUITEMS, Quetta, Pakistan; 2 Institute of Human Genetics, University of Cologne, Cologne, Germany; 3 Institute for Genetics, University of Cologne, Cologne, Germany

## Abstract

**Background:**

Hereditary cataracts are most frequently inherited as autosomal dominant traits, but can also be inherited in an autosomal recessive or X-linked fashion. To date, 12 loci for autosomal recessive cataracts have been mapped including a locus on chromosome 16q22 containing the disease-causing gene *HSF4 *(Genbank accession number NM_001040667). Here, we describe a family from Pakistan with the first nonsense mutation in *HSF4 *thus expanding the mutational spectrum of this heat shock transcription factor gene.

**Methods:**

A large consanguineous Pakistani family with autosomal recessive cataracts was collected from Quetta. Genetic linkage analysis was performed for the common known autosomal recessive cataracts loci and linkage to a locus containing *HSF4 *(OMIM 602438) was found. All exons and adjacent splice sites of the heat shock transcription factor 4 gene (*HSF4*) were sequenced. A mutation-specific restriction enzyme digest (H*ph*I) was performed for all family members and unrelated controls.

**Results:**

The disease phenotype perfectly co-segregated with markers flanking the known cataract gene HSF4, whereas other autosomal recessive loci were excluded. A maximum two-point LOD score with a Zmax = 5.6 at θ = 0 was obtained for D16S421. Direct sequencing of HSF4 revealed the nucleotide exchange c.1213C > T in this family predicting an arginine to stop codon exchange (p.R405X).

**Conclusion:**

We identified the first nonsense mutation (p.R405X) in exon 11 of *HSF4 *in a large consanguineous Pakistani family with autosomal recessive cataract.

## Background

Congenital cataracts show considerable clinical and locus heterogeneity and represent one of the major causes of vision loss world-wide [1,2]. Cataracts can be isolated or can occur in association with a large number of different metabolic diseases or genetic syndromes [3]. Nonsyndromic congenital cataracts tend to be highly penetrant as Mendelian traits with autosomal dominant more common than autosomal recessive forms. Nonsyndromic congenital cataract has an estimated frequency of 1–6 per 100,000 live births [4]. To date, loci for 28 autosomal dominant and 12 autosomal recessive forms of congenital cataracts have been mapped. Out of 12 autosomal recessive loci, mutations in eight genes have been identified [5-16]. Of those families with inherited cataracts for whom the mutant gene is known, about half show mutations in genes encoding for crystallins, about a quarter have mutations in genes coding for connexins, with the remainder divided among the genes encoding the heat shock transcription factor-4 (*HSF4*), aquaporin-0 (*AQP0*, MIP), and beaded filament structural protein-2 (*BFSP2*) [3].

Here, we report a large consanguineous family from Pakistan with eight affected individuals affected by autosomal recessive cataracts. Consanguineous marriages are a common practice in the Pakistani society and 60% of marriages are reported to be within families [17]. Such families have been instrumental for mapping disease loci and for identification of causative genes and mutations in especially autosomal recessive disorders. During linkage analysis for known recessive cataracts loci, we showed co-segregation of the disease phenotype with markers *D16S397 *(85.94 cM corresponding to position 65,295,710 bp in NCBI build 36.1), *D16S3086 *(85.94 cM; 65,493,383 bp) and *D16S421 *(85.94 cM; 65,853,130 bp). A maximum two-point LOD score of 5.6 at recombination fraction θ = 0 was obtained for marker *D16S421*. As the critical interval contained the *HSF4 *gene (65,754,789 – 65,761,349 bp) previously reported to cause autosomal dominant or autosomal recessive cataracts, sequence analysis of *HSF4 *was performed which identified a novel nonsense mutation (p.R405X) in exon 11 as the cause of the disease in this family.

## Methods

### Family enrollment and clinical evaluation

**Figure 1:**
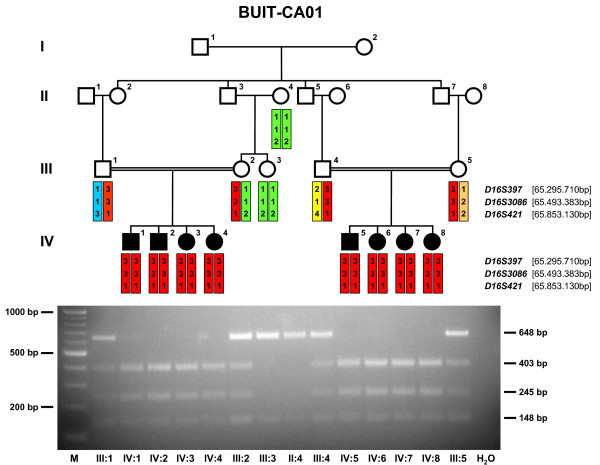
**Pedigree structure and linkage and restriction enzyme digest analysis of family BUIT-CA01 with autosomal recessive congenital cataracts**. The marker alleles and haplotypes for three microsatellites covering the *HSF4 *locus on chromosome 16, are shown beneath the pedigree symbols. The location of markers according to NCBI build 36.1 is given in brackets behind the marker names (the *HSF4 *gene is located at 65, 754, 789–65, 761, 349 bp). An allele-specific restriction enzyme (H*ph*I) digest demonstrates homozygous wild type alleles (648 bp and 148 bp) in individuals II:4 and III:3 and homozygous mutant alleles (403 bp, 248 bp and 148 bp) in the affected individuals IV:1, IV:2, IV:3, IV:4, IV:5, IV:6, IV:7 and IV:8, whereas the parents (III:1, III:2, III:4 and III:5) each carrying one WT and one mutant allele. M = 100 bp-ladder, H_2_O = PCR water control.

The family (BUIT-CA01, Figure 1) was enrolled from Laytton Rahmatullah Benevolent Trust (LRBT) Hospital Quetta, Pakistan. Approval for this study was obtained from the IRB at Faculty of Biotechnology and Informatics, BUITEMS, Quetta Pakistan. Written informed consent was obtained from all the 14 participating subjects or their guardians with the tenets of the Declaration of Helsinki. A detailed medical history was obtained by interviewing all family members. Cataracts in affected individuals were either present at birth or developed during infancy. All affected individuals had undergone cataract surgery so that the lens phenotype could not be ascertained in more detail. During clinical examinations of affected individuals (IV:1, IV:2, IV:3, IV:4, IV:5, IV:6, IV:7 and IV:8), there was no other ocular or systemic abnormalities or symptoms except cataracts. Neither of the parents (III:1, III:2, III:4 and III:5) and none of the unaffected relatives (II:4 and III:3) had any evidence of cataracts.

### Linkage analysis

Genomic DNA was extracted from blood samples obtained from all participating family members (8 affected and 6 unaffected individuals) by a standard protocol [18]. All reported common autosomal recessive cataract loci were genotyped by selecting 2–3 STR markers for each candidate interval (additional file 1). Linkage analysis was performed by selecting 4 affected (IV:1, IV:2, IV:3, IV:4) and 3 unaffected individuals (III:1, III:2, III:3). PCR was performed with an initial inactivation step at 94°C for 3 min following by 30 cycles of denaturation at 94°C for 30 s, annealing at 54°C for 30 s and extension at 72°C for 40 s with a final extension at 72°C for 10 min. Amplified products were run on 6% denaturing polyacrylamide gels and were visualized by silver staining. For linkage analysis, the Marshfield genetic map  was consulted for marker order and map distances, physical positions correspond to NCBI build 36.1 as given in the UCSC Genome Browser . Two-point Lod scores were calculated by using the FASTLINK program in the easyLINKAGE software package [19]. The disease was regarded as fully penetrant and the disease allele frequency was set at 0.0001. Meiotic recombination frequencies were assumed to be equal for males and females. Allele frequencies of microsatellite markers were determined by genotyping 100 unaffected individuals from the Pakistani population.

### Mutational Analysis

Primers for PCR amplification and subsequent sequencing of *HSF4 *(NM_001040667 coding for the larger isoform B of this alternatively spliced gene) were designed by using software at the primer3 web site  to flank all exon-intron boundaries. Exons amplification, sequencing reactions and mutational analysis were performed by standard protocols. DNA sequencing was performed using BigDye version 2.1 and an ABI 3100 sequencing apparatus (Applied Biosystem, Foster City, CA). All exons and exon/intron border regions were sequenced and aligned to the GenBank reference sequence. Reactions were performed in 10 ul volumes according to the manufactures' protocols. PCR reactions were analyzed by 2% agarose gel electrophoresis by staining with ethidiumbromide before sequencing. Thermal cycling conditions were an initial inactivation step at 95°C for 5 min followed by first 10 cycles as touch town PCR (with annealing 68°C to 58°C) and additional 27 cycles with denaturation at 95°C for 30 s, annealing at 57°C for 30 s and extension at 72°C for 1 min with a final extension at 72°C for 10 min. The identified mutation was analyzed by an allele-specific restriction enzyme (H*ph*I) digest. This digest was performed in a reaction volume of 20 ul, using 4 ul PCR product and 5 units of the enzyme. The digested PCR products were analyzed by 2% agarose gels and the DNA was visualized by staining with ethidiumbromide. The expected fragments sizes for the homozygous wild-type allele were 648 bp and 148 bp, whereas homozygous mutant alleles should show products at 403 bp, 245 bp and 148 bp. To further support the pathogenicity of the mutation, altogether 167 control samples (91 German and 76 Pakistani control individuals) were also analyzed by the above-mentioned diagnostic digest.

## Results

**Figure 2:**
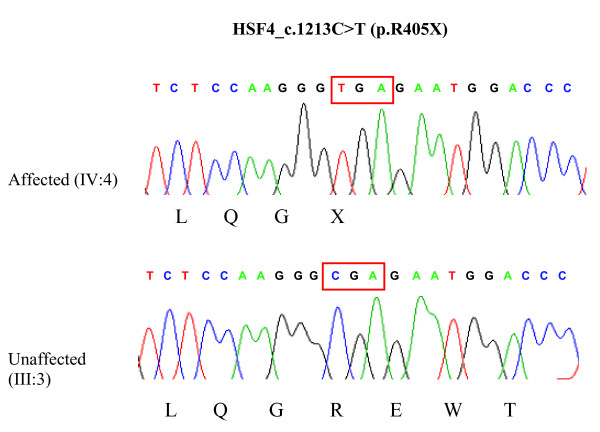
**Results of sequence analysis of exon 11 of the *HSF4 *gene**. A homozygous C > T transition was found at nucleotide 1213 (according to Genbank entry NM_001040667.2 encoding isoform B) in the affected individual (IV:4), predicted to change the amino acid arginine into a stop codon, while an unaffected family member (III:3) was homozygous for the WT-allele.

This family (Fig. 1) originated from Afghanistan and migrated to Quetta-Pakistan in the late 1980s. Cataracts in affected individuals were either present at birth or developed during infancy. By locus-specific linkage analysis, the disease phenotype was shown to perfectly co-segregate with flanking markers to the already known causative gene *HSF4 *(OMIM 602438). A highly significant maximum two-point LOD score was obtained at *D16S421 *(Zmax = 5.6 at θ = 0) (table 1). PCR amplification and subsequent direct sequencing of the coding exons of *HSF4 *(based on the largest coding Genbank entry with the accession number NM_001040667.2) revealed the novel nucleotide exchange c.1213C > T in exon 11 of *HSF4 *in the family BUIT-CA01 (Fig. 2). This mutation was not present in any of the unaffected individuals neither of this family nor in altogether 334 control alleles of Pakistani and German healthy control samples. This transition is predicted to change the amino acid arginine into a stop codon (p.Arg405X in isoform B [NP_001035757.1] corresponding to p.Arg375X in isoform A [NP_001529.2]) (Fig 2). A mutation-specific restriction enzyme (H*ph*I) digest confirmed homozygosity for the mutant allele in all eight affected individuals (IV:1, IV:2, IV:3, IV:4, IV:5, IV:6, IV:7 and IV:8; bands of 403 bp, 245 bp and 148 bp, Fig. 1) whereas homozygous wild type alleles were shown in individual II:4 and III:3 (bands of 648 bp and 148 bp; Fig. 1). The four parents (III:1, III:2, III:4 and III:5) showed heterozygosity for one WT allele (648 bp and 148 bp) and one mutant allele (403 bp, 245 bp and 148 bp; Fig. 1).

**Table 1:** Two-point LOD score obtained between the disease in family BUIT-CA01 and each marker locus

	**LOD score values at θ**				
**Marker**	**0.0**	**0.1**	**0.2**	**0.3**	**0.4**
D16S397	5.4	4.3	3.2	2.0	0.8
D16S3086	5.4	4.3	3.2	2.0	0.8
D16S421	5.6	4.5	3.4	2.2	1.0

Markers allele frequencies were determined by typing 100 normal ethnically matched samples, no. of alleles for marker D16S397 were 6 with frequencies (0.12, 0.20, 0.17, 0.11, 0.29 and 0.11), for marker D16S421, no. of alleles were 8 with frequencies (0.16, 0.21, 0.11, 0.17, 0.04, 0.14, 0.10, 0.07) and for marker D16S3086, no of alleles were found to be 7 with allele frequencies (0.13, 0.05, 0.26, 0.18, 0.09, 0.17, 0.12).

## Discussion

*HSF4 *(OMIM 602438) belongs to the family of heat shock transcription factors that regulate the expression of heat shock proteins in response to different cellular stresses, such as oxidants, heavy metals, elevated temperature, and bacterial or viral infections. Two isoforms, *HSF4a *and *HSF4b*, resulting from two alternative spliced regions in exons 8 and 9 have been reported [20,21]. *HSF4a *actively represses transcription of other heat shock factor genes by directly binding to the heat shock elements. *HSF4b*, which has 30 additional amino acids, acts as an activator of transcription. It has been demonstrated that the additional 30 amino acids are responsible for this activity [22,23].

Here, we report the first *HSF4 *nonsense mutation in a large Afghani/Pakistani family. DNA sequencing revealed the transition c.1213C > T (p.Arg405X) in exon 11. This mutation is predicted to cause a premature termination, presumably resulting in a complete loss of function of the aberrant *HSF4 *protein in affected homozygotes. This causes a severe phenotype with autosomal recessive congenital cataracts. Indeed, the *HSF4 *gene that has been reported responsible both for autosomal dominant and autosomal recessive cataracts. The association of the *HSF4 *gene with two different modes of inheritance for congenital cataracts can at least in part be explained by the location and severity of the mutations. Interestingly, all known dominant mutations in *HSF4 *lie within the α-helical DNA binding domain, whereas the recessive mutations lie outside this highly conserved functional domain. To-date, only eight different mutations in *HSF4 *have been reported, three of them are causing autosomal dominant cataracts, three are causing autosomal recessive cataracts, and two mutations were found in sporadic cases. In addition to this novel nonsense mutation in exon 11, the already known mutations in *HSF4 *comprise six missense alterations, one deletion, and one splice site mutation (Human Mutation Gene Database, ). Exon 11 is encoding the so called DHR domain of the *HSF4 *protein, the importance of which was already demonstrated by identifying a 5'splice site mutation in intron 12 causing skipping of exon 12 in a large Tunisian family [8]. Two previously identified mutations (p.Arg175Pro and c.595_599delGGGcc) in Pakistani families showed recessive cataracts and are located in the HR-A and HR-B domains of the *HSF4 *protein again demonstrating the extreme genetic heterogeneity of the Pakistani population [24]. Historically, Pakistan has experienced not only internal migrations across the Indus valley but also successive waves of migrations from northwest. In more recent times, the region experienced massive migrations of refugees from Afghanistan. So, the Pakistani society is composed of many ethnic groups and many of these are common to populations of India, Afghanistan, Bangladesh and Iran. Concerning the clinical phenotype caused by mutations in this transcription factor, it is unclear, why mutations in *HSF4 *that is also expressed in tissues including the heart, muscle, lung and brain should cause only nonsyndromic cataracts [3,8] and more comprehensive studies will be needed to answer this question.

## Conclusion

As a conclusion, we have shown the first nonsense mutation in *HSF4 *causing autosomal recessive cataracts in a large consanguineous family from Pakistan. The identification of this and additional mutations enables proper genetic diagnostics and counseling in affected families and may lead to a better understanding of the structure and function of *HSF4 *in health and disease.

## Competing interests

The authors declare that they have no competing interests.

## Authors' contributions

NS performed the clinical ascertainment and linkage analysis, IG performed the mutation screening, NK helped in linkage analysis and clinical evaluation, AMC provided funds for this study, CK analyzed and supervised the mutation identification, JA designed, and supervised the study, CK and JA drafted the manuscript. All authors read and approved the final manuscript.

## Pre-publication history

The pre-publication history for this paper can be accessed here:



## Supplementary Material

Additional file 1 **Results of linkage analysis for the markers used to analyze other cataracts loci.** Click here for file
