# Supplementary material for: A novel HSF4 gene mutation (p.R405X) causing autosomal recessive congenital cataracts in a large consanguineous family from Pakistan
Source: BMC Med Genet. 2008 Nov 11;9:99. doi: 10.1186/1471-2350-9-99 (PMC2592245; doi:10.1186/1471-2350-9-99)
Supplement: Additional file 1 — Results of linkage analysis for the markers used to analyze other cataracts loci. [file 1471-2350-9-99-S1.doc]

**Additional table 1: Results of linkage analysis for the markers used to analyze other cataracts loci**

| Chromosome /gene | Markers | Marshfield position | III:1 | III:2 | IV:1 | IV:2 | IV:3 | IV:4 | III:3 | LOD score  θ = 0 |
| --- | --- | --- | --- | --- | --- | --- | --- | --- | --- | --- |
| *GJA8* | D1S1156 | 155.89 cM | 2 2 | 1 3 | 2 1 | 2 3 | 1 2 | 2 3 | 3 2 | -3.86 |
|  | D1S2612 | 155.89 cM | 2 3 | 3 1 | 2 3 | 3 1 | 3 3 | 2 1 | 1 2 | -3.76 |
| *GCNT2* | D6S940 | 16.84 cM | 2 1 | 1 3 | 2 3 | 2 1 | 1 3 | 1 1 | 2 3 | -5.98 |
|  | D6S470 | 18.22 cM | 2 3 | - - | 2 1 | 2 3 | 3 1 | 3 3 | 2 1 | -5.99 |
| *LIM2* | D19S553 | 81.51 cM | 2 2 | 1 3 | 2 3 | 2 1 | 2 3 | 2 1 | 1 4 | -4.53 |
|  | D19S402 | 83.19 cM | 1 3 | 3 2 | 2 2 | 1 3 | 1 2 | 2 3 | 3 1 | -4.48 |
| *BFSP1* | D20S112 | 39.25 cM | 2 1 | 3 2 | 2 2 | 1 3 | 1 2 | 2 3 | 3 1 | -2.98 |
|  | D20S470 | 39.25 cM | 1 2 | 2 2 | 1 2 | 2 2 | 2 2 | 1 2 | 2 3 | -2.99 |
| *CRYAA* | D21S1411 | 51.49 cM | 2 2 | 1 3 | 2 1 | 2 3 | 1 2 | 2 3 | 3 2 | -3.31 |
|  | D21S1890 | 52.50 cM | 2 3 | 3 1 | 2 3 | 3 1 | 3 3 | 2 1 | 1 2 | -3.28 |
|  | D21S1885 | 52.50 cM | 2 1 | 1 3 | 2 3 | 2 1 | 1 3 | 1 1 | 2 3 | -3.15 |
| *9q13-q22* | D9S167 | 83.41 cM | 2 1 | 3 2 | 2 2 | 1 3 | 1 2 | 2 3 | 3 1 | -4.91 |
|  | D9S152 | 84.90 cM | 1 2 | 2 2 | 1 2 | 2 2 | 2 2 | 1 2 | 2 3 | -4.88 |
| *CRYBB3* | D22S1028 | 21.47 cM | 2 3 | 3 1 | 2 1 | 2 3 | 3 1 | 3 3 | 2 1 | -5.91 |
|  | D22S925 | 21.47 cM | 2 2 | - - | 2 3 | 2 1 | 2 3 | 2 1 | 1 4 | -5.86 |
|  | D22S926 | 21.47 cM | 1 3 | 3 2 | 2 2 | 1 3 | 1 2 | 2 3 | 3 1 | -5.81 |
